# Incidence and risk of pancreatic cancer in patients with chronic pancreatitis: defining the optimal subgroup for surveillance

**DOI:** 10.1038/s41598-022-26411-8

**Published:** 2023-01-03

**Authors:** Hyo Suk Kim, Tae-Geun Gweon, Sang Hi Park, Tae Ho Kim, Chang Whan Kim, Jae Hyuck Chang

**Affiliations:** 1 Department of Internal Medicine, College of Medicine, The Catholic University of Korea, Seoul, Republic of Korea; 2 Institute of Clinical Medicine Research, College of Medicine, The Catholic University of Korea, Bucheon, Korea; 3 Division of Gastroenterology, Department of Internal Medicine, Bucheon St. Mary’s Hospital, College of Medicine, The Catholic University of Korea, 327, Sosa-Ro, Wonmi-Gu, Bucheon-Si, Gyeonggi-Do 14647 Republic of Korea

**Keywords:** Pancreatic cancer, Pancreatitis

## Abstract

We aimed to present the incidence and risk factors for pancreatic cancer in a multicenter retrospective cohort of patients with chronic pancreatitis (CP). Patients with ICD-10 codes for CP (K86.0, K86.1) who underwent abdominal CT or MRI between January 2010 and December 2021 in seven academic hospitals were analyzed. After exclusions, we identified 727 patients with definite CP with a median follow-up of 3.6 years (range 1.0‒12.9). During 3290 person-years of observation, pancreatic cancers were diagnosed in 16 patients (2.20%, 0.49% per year) after a median follow-up of 2.4 years (range 1.4‒6.6), with an age- and sex-standardized incidence ratio of 18.1 (95% CI 10.4‒29.5). The underlying CPs in the 16 pancreatic cancers were classified as chronic obstructive pancreatitis (10, 63%), chronic obstructive and calcifying pancreatitis (4, 25%), chronic calcifying pancreatitis (1, 6%), and autoimmune pancreatitis (1, 6%). Factors associated with pancreatic cancer development included age (HR 4.830, *p* = 0.006), parenchymal calcification (HR 0.213, *p* = 0.003), pancreatic duct stricture (HR 2.706, *p* = 0.048), and serum CA 19‒9 level (HR 3.567, *p* = 0.014). After adjustment, age over 60 years (HR 4.540, *p* = 0.009) and serum CA 19‒9 levels greater than 100 U/mL (HR 3.528, *p* = 0.015) were independent risk factors for pancreatic cancer.

## Introduction

Pancreatic cancer is one of the cancers with the worst prognosis and has the lowest 5-year survival rate among all cancers, at around 10%. The cancer death rates of lung cancer, prostate cancer, breast cancer, and colon cancer, which have higher cancer death rates than pancreatic cancer in the United States, have all been declining over times. However, the cancer death rate for pancreatic cancer has increased slightly (by 0.2% per year) since the mid-2000s according to the American Cancer Society, Cancer Facts & Figures 2022. The mortality of pancreatic cancer is predicted to increase over time, reaching the second-highest overall cancer mortality rate in the United States by 2030^1^. Although much effort has been made to improve the low survival rate of patients with pancreatic cancer, no tangible progress has been made yet. The most effective method for survival improvement to date is the early detection of pancreatic cancer through screening tests. Therefore, it is important to identify and monitor high-risk groups for pancreatic cancer.

Patients with chronic pancreatitis (CP) is one of the major risk groups for pancreatic cancer, and the relative risk of pancreatic cancer in patients with CP has been reported to be as high as 7.6‒68.1 times^2–6^. The incidence of pancreatic cancer in patients with CP has been mainly investigated in Western countries, where it was reported to be 1.0‒2.6%^4,7–10^. In a small number of studies in Asia, the incidence of pancreatic cancer was found to be 0.9‒2.9%^11–13^. The incidence and standardized incidence ratio (SIR) of pancreatic cancer in patients with CP have rarely been reported in South Korea. In the 2020 International Consensus Guidelines on Surveillance for Pancreatic Cancer in CP, it was not recommended to monitor for pancreatic cancer in all sporadic CP cases because the prevalence of pancreatic cancer is not high enough^14^. According to international guidelines, patients with CP accompanied by serine protease 1 gene (*PRSS1*) mutation are recommended to undergo regular follow-up for pancreatic cancer detection. However, other conditions required for surveillance in patients with CP were not presented, and additional related research is still necessary. To date, several studies have suggested that risk factors for pancreatic cancer in CP include newly developed diabetes, obesity, pancreatic duct dilatation, old age, and current smoker^3,8–11^. However, these risk factors need to be verified, and risk groups of patients with CP for surveillance should be selected using risk factors for pancreatic cancer.

Therefore, we aimed to determine the incidence of pancreatic cancer in patients with CP and to obtain age- and sex-standardized incidence ratios (SIRs) using the incidence of pancreatic cancer in the general population. In addition, we wanted to present subgroups of patients with CP who should be followed up for pancreatic cancer by analyzing which factors are related to the development of pancreatic cancer in CP and the incidence of pancreatic cancer according to risk factors.

## Results

### Study population

A total of 727 patients with definite CP were evaluated, and the median follow-up period was 1,311 days (range 368‒4698 days) (Table 1). There were 588 (80.9%) males and 139 (19.1%) females with a median age of 56.5 years (range 18‒93). More than half (61.8%) had diabetes mellitus, and a lower BMI (< 20, 36.0%) was much common than a higher BMI (> 25, 16.3%). Two radiologic studies were performed to evaluate the pancreas: CT (mean 3.8 times per patient) and MRI (mean 0.6 times per patient). A total of 512 patients (70.4%) had parenchymal calcification, 269 (37.0%) had pancreatic duct stones, 152 (20.9%) had pancreatic duct stricture, 24 (3.3%) had groove pancreatitis, and 29 (4.0%) had autoimmune pancreatitis**.**

**Table 1:** Baseline characteristics of patients.

Characteristics	Total (%)	Chronic pancreatitis without pancreatic cancer (%)	Chronic pancreatitis with pancreatic cancer (%)	*p* value
Total	727	711 (97.8)	16 (2.2)	
**Sex**				0.206
Male	588 (80.9)	577 (81.1)	11 (68.8)	
Female	139 (19.1)	134 (18.9)	5 (31.2)	
**Age at diagnosis, years**				0.043
< 50	218 (30.0)	216 (30.4)	2 (12.5)	
50‒59	219 (30.1)	217 (30.5)	2 (12.5)	
60‒69	173 (23.8)	166 (23.4)	7 (43.8)	
70‒79	93 (12.8)	89 (12.5)	4 (25.0)	
≥ 80	24 (3.3)	23 (3.2)	1 (6.2)	
Follow-up period, median years (IQR)	3.59 (2.09‒6.31)	3.61 (2.13‒6.40)	2.36 (1.70‒3.95)	0.060
**BMI**				0.740
< 20	230 (36.0)	223 (35.8)	7 (43.8)	
20‒25	305 (47.7)	299 (48.0)	6 (37.5)	
25‒30	88 (13.8)	85 (13.7)	3 (18.7)	
≥ 30	16 (2.5)	16 (2.6)		
**DM**				0.311
No	278 (38.2)	274 (38.5)	4 (25.0)	
Yes	449 (61.8)	437 (61.5)	12 (75.0)	
**Acute exacerbation of pancreatitis**				0.031
No	576 (79.2)	567 (79.8)	9 (56.2)	
Yes	151 (20.8)	144 (20.2)	7 (43.8)	
**Groove pancreatitis**				0.419
No	702 (96.7)	687 (96.8)	15 (93.8)	
Yes	24 (3.3)	23 (3.2)	1 (6.2)	
**Autoimmune pancreatitis**				0.482
No	698 (96.0)	683 (96.1)	15 (93.8)	
Yes	29 (4.0)	28 (3.9)	1 (6.2)	
**Parenchymal calcification**				0.009
No	215 (29.6)	205 (28.8)	10 (62.5)	
Yes	512 (70.4)	506 (71.2)	6 (37.5)	
**Pancreatic duct dilatation**				0.576
No	205 (28.2)	202 (28.4)	3 (18.8)	
Yes	522 (71.8)	509 (71.6)	13 (81.2)	
**Pancreatic duct stone**				0.063
No	458 (63.0)	444 (62.5)	14 (87.5)	
Yes	269 (37.0)	267 (37.6)	2 (12.5)	
**Pancreatic duct stricture**				0.054
No	575 (79.1)	566 (79.6)	9 (56.2)	
Yes	152 (20.9)	145 (20.4)	7 (43.8)	
**Irregular pancreatic duct**				1.000
No	618 (85.0)	604 (85.0)	14 (87.5)	
Yes	109 (15.0)	107 (15.0)	2 (12.5)	
**Pancreatic cyst**				1.000
No	509 (70.1)	498 (70.0)	11 (68.8)	
Yes	217 (29.9)	212 (30.0)	5 (31.2)	
**CBD stricture**				0.114
No	577 (79.4)	567 (79.8)	10 (62.5)	
Yes	150 (20.6)	144 (20.2)	6 (37.5)	
**P duct stent placement**				0.621
No	674 (92.8)	658 (92.7)	16 (100.0)	
Yes	52 (7.2)	52 (7.3)	0 (0.00)	
**Previous pancreatobiliary surgery**				0.076
No	651 (89.5)	639 (89.9)	12 (75.0)	
Yes	76 (10.5)	72 (10.1)	4 (25.0)	
**CA 19‒9 level**				0.001
≥ 100 U/mL	54 (8.7)	49 (8.1)	5 (31.3)	
< 100 U/mL	568 (91.3)	557 (91.9)	11 (68.8)	

*IQR* interquartile range, *BMI* body mass index, *CBD* common bile duct, *P duct* pancreatic duct.

### Comparison of CP patients with and without pancreatic cancer

During 3,290 person-years of observation, pancreatic cancers were diagnosed in 16 patients (overall 2.20% and 0.49% per year) after a median follow-up of 2.4 years (range 1.4‒6.6). Compared to patients without pancreatic cancer, CP patients with pancreatic cancer were older (*p* = 0.043), had more frequent exacerbated pancreatitis (*p* = 0.031), and had higher CA 19‒9 levels (*p* = 0.001). However, parenchymal calcification was lower in the cancer group (*p* = 0.009). Patients aged 60 or older represented 75.0% of the pancreatic cancer group and 39.1% of the non-pancreatic cancer group (*p* = 0.008).

### Characteristics of CP in patients with pancreatic cancer

The details of the 16 patients with pancreatic cancer are described in Table 2. The median age of patients with pancreatic cancer was 66.4 years (range 43‒82 years), 11 (69%) were male, the median BMI was 20.1 (range 16.0‒27.3), 12 (75%) had diabetes, and 8 (50%) had pancreatic atrophy. Five (31%) had pancreatic cysts and 2 (13%) had pancreatic duct stones. Regarding the classifications of underlying CP, 10 of the 16 patients who developed pancreatic cancer (63%) had chronic obstructive pancreatitis, 4 (25%) had chronic obstructive and calcifying pancreatitis, 1 (6%) had chronic calcifying pancreatitis, and 1 (6%) had autoimmune pancreatitis. Therefore, pancreatic cancer appears to be more likely to occur in patients with chronic obstructive pancreatitis than in patients with chronic calcifying pancreatitis.

**Table 2:** Details of pancreatic cancers in patients with chronic pancreatitis.

No	Age	Sex	DM	BMI	Smoking	Classification of pancreatitis	Time to cancer (mo)	Progression
1	68	M	Yes	20.2	No	Chronic obstructive	17	Pancreatic duct stricture in the head → 1.2 × 2.3 cm mass in the body
2	62	M	Yes	17.9	Unclear	Chronic obstructive groove	19	Groove pancreatitis and pancreatic duct stricture in the head infiltrative mass in the head
3	71	F	Yes	24.6	No	Chronic obstructive	20	Pancreatic duct stricture in the neck → 4 cm pancreatic mass with lung metastasis
4	77	M	Yes	20.1	No	Chronic obstructive	20	Diffuse parenchymal atrophy with mild dilatation of the pancreatic duct and small cystic lesions → 2.4 × 2 cm mass in the body
5	63	F	Yes	27.0	Unclear	Chronic obstructive	23	Pancreatic duct stricture and parenchymal atrophy change → 3.8 × 2.6 cm infiltrative mass in the body and tail with peripancreatic infiltration
6	54	M	Yes	20.7	No	Chronic obstructive	25	Severe pancreatic parenchymal atrophy with fatty replacement → 2.1 × 1.5 cm mass in the head with diffuse pancreatic duct dilatation
7	59	M	Yes	19.1	Yes	Chronic obstructive and calcifying	25	Mild pancreatic parenchymal atrophy, pancreatic duct dilatation, and multiple tiny calcifications → 2.6 × 2.2 cm mass in the head
8	66	M	Yes	17.4	No	Chronic obstructive and calcifying	28	Multiple calcifications and dilated distal pancreatic duct → 1.7 × 2.3 cm mass in the body
9	66	F	No	23.4	No	Autoimmune	29	Diffuse enlargement with heterogeneous enhancement throughout the pancreas → multiple peritoneal and omental masses, peritoneal carcinomatosis
10	61	M	Yes	26.8	No	Chronic calcifying	37	Calcifications in the uncinate process → 17 mm mass in the head with multiple liver metastasis
11	82	F	No	27.3	No	Chronic obstructive and calcifying	43	Multiple calcifications in the pancreas and pancreatic duct stricture in the body → 1 cm mass in the body
12	69	M	Yes	16.0	No	Chronic obstructive	44	Distal pancreatectomy due to pancreatic duct stone → 4.5 × 4.0 cm mass in the head
13	47	M	Yes	19.2	Yes	Chronic obstructive	49	Pancreatic duct stricture in the head → 1.2 × 1.3 cm mass in the head
14	77	M	No	21.6	No	Chronic obstructive	65	Pancreatic duct stones, parenchyma atrophy, and CBD and IHD dilatation → 7 cm mass in the head
15	77	F	No	17.6	No	Chronic obstructive and calcifying	70	Diffuse pancreatic duct dilatation with parenchymal calcification → 1.4 cm mass in the head
16	43	M	Yes	16.1	Yes	Chronic obstructive	79	Pancreatic duct stricture in the head, atrophy in the tail, and diffuse dilatation in bile duct → heterogeneous enhancement in the head

*DM* diabetes mellitus, *BMI* body mass index.

Ten patients (13.8%) had a family history of pancreatic cancer, and none in the group with pancreatic cancer had a family history of pancreatic cancer. One patient had hereditary pancreatitis and three family members of one patient had a pancreatitis history. Fifty-two patients (7.2%) had a family history of cancer other than pancreatic cancer, 2 patients (12.5%) in the group with pancreatic cancer and 50 patients (7.0%) in the group without pancreatic cancer. There was no significant difference between the two groups.

### Incidence of pancreatic cancer during follow-up

**Figure 1:**
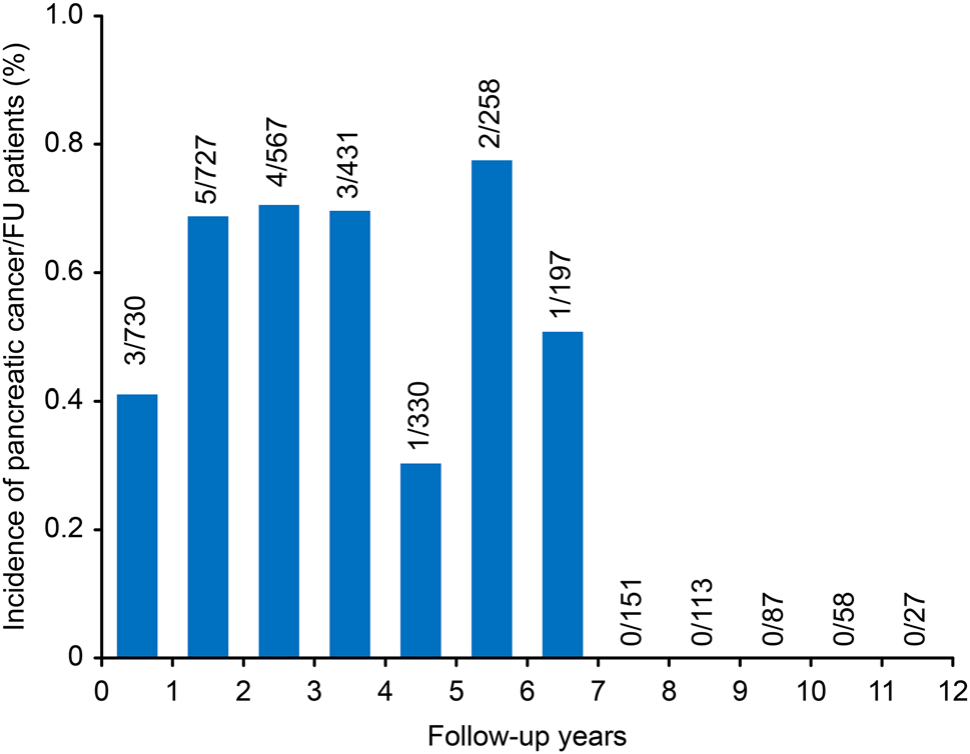
Incidence of pancreatic cancers during follow-up.

**Figure 2:**
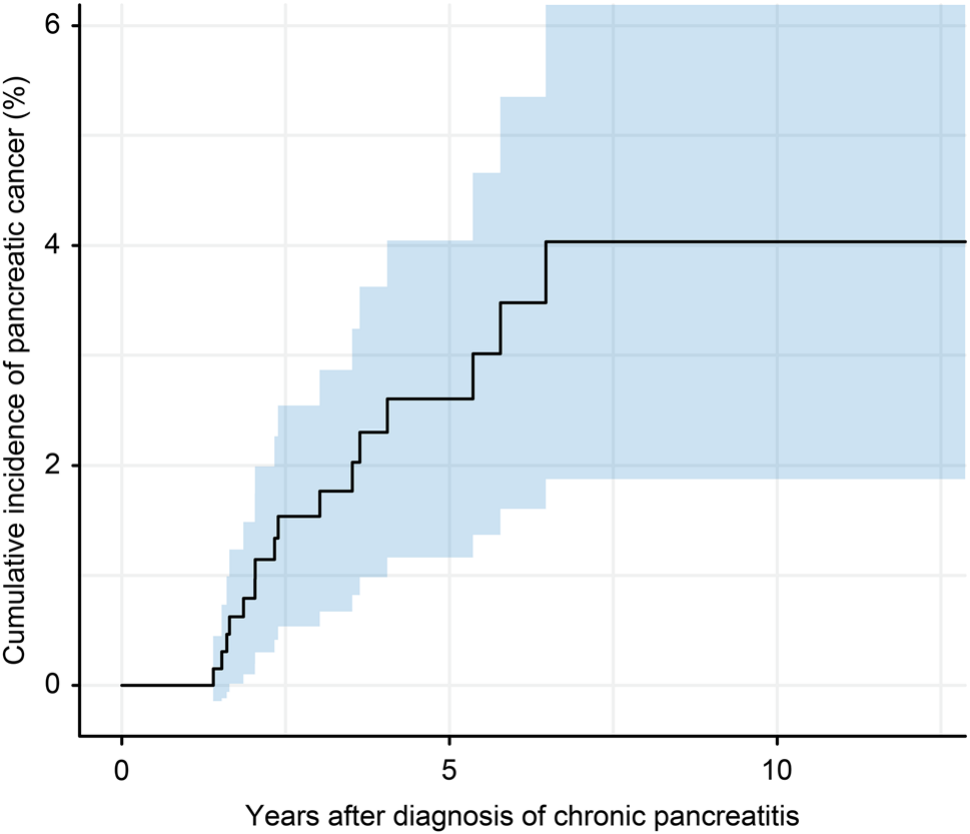
Cumulative incidence of pancreatic cancer. The blue area is the 95% confidence interval.

When the 16 pancreatic cancer patients were evaluated based on detection time, the incidence during each year was 0.49% (range 0.30‒0.77%) (Fig. 1). The incidence remained relatively steady until 6.5 years, and pancreatic cancer did not develop thereafter. The cumulative incidences of pancreatic cancer in patients with CP were 1.0% at 2 years and 2.6% at 4 years and reached 4.0% at 6.5 years according to the Kaplan‒Meier analysis (Fig. 2).

### Standardized incidence ratio of pancreatic cancer in CP

The age- and sex-SIR of pancreatic cancer in patients with CP was 18.1 (95% CI 10.4‒29.5) (Table 3). According to age groups, HR was highest at 59.7 (95% CI 34.1‒97.0) in the 40‒49 age group and was 23.6 (95% CI 13.5‒38.3) in the 60‒69 age group.

**Table 3:** Age- and sex-standardized incidence ratio of pancreatic cancers in chronic pancreatitis.

Age	General male incidence rate (per 100,000 person-years)	General female incidence rate (per 100,000 person-years)	Number of patients with CP	Expected No	Observed No	HR	95% CI
20‒29	0.3	0.9	18	0.0006	0 (0.0%)	0	
30‒39	1.2	1.6	55	0.0030	0 (0.0%)	0	
40‒49	4.8	3.5	144	0.0335	2 (1.4%)	59.7	34.1‒97.0
50‒59	17.2	10.4	218	0.1630	2 (0.9%)	12.3	7.01‒20.0
60‒69	41.0	27.3	175	0.2971	7 (4.0%)	23.6	13.5‒38.3
70‒79	81.0	59.9	93	0.2934	4 (4.3%)	13.6	7.79‒22.1
≥ 80	115.5	83.1	24	0.0915	1 (4.1%)	10.9	6.25‒17.8
Total	19.1	16.8	727	0.8821	16 (2.2%)	18.1	10.4‒29.5

*CP* chronic pancreatitis, *HR* hazard ratio, *CI* confidence interval.

### Risk factors for pancreatic cancer in patients with CP

**Figure 3:**
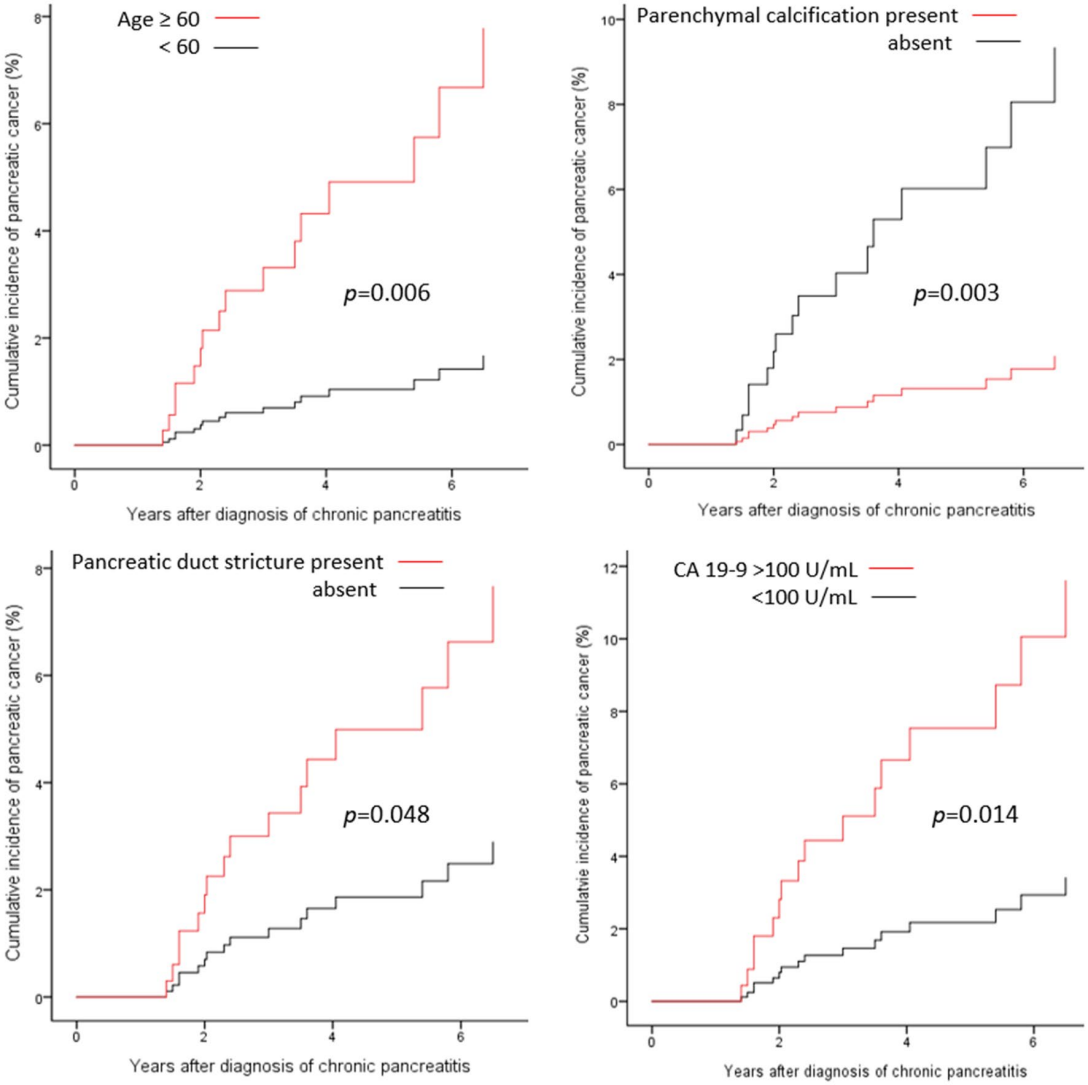
Risk factors for pancreatic cancer in patients with chronic pancreatitis: Cox regression analysis.

Risk factors for pancreatic cancer in patients with CP were analyzed using Cox regression analysis (Table 4). Factors associated with pancreatic cancer development included age (HR 4.830, 95% CI 1.556‒14.99, *p* = 0.006), parenchymal calcification (HR 0.213, 95% CI 0.077‒0.586, *p* = 0.003), pancreatic duct stricture (HR 2.706, 95% CI 1.007‒7.271, *p* = 0.048), and CA 19‒9 level (HR 3.567, 95% CI 1.294‒9.831, *p* = 0.014) (Fig. 3). After adjusting for variables, age (HR 4.540, 95% CI 1.464‒14.08, *p* = 0.009) and CA 19‒9 level (HR 3.528, 95% CI 1.282‒9.710, *p* = 0.015) were identified as independent risk factors for pancreatic cancer.

**Table 4:** Risk factors for pancreatic cancer in patients with chronic pancreatitis: Cox regression analysis.

Characteristics	Univariate			Multivariate		
	HR	95% CI	*p* value	HR	95% CI	*p* value
Sex	2.049	0.712‒5.899	0.184			
Age (< 60 vs. ≥ 60)	4.830	1.556‒14.99	0.006	4.540	1.464‒14.08	0.009
BMI (< 20 vs. ≥ 20)	0.508	0.267‒1.923	0.639			
DM	1.720	0.555‒5.334	0.348			
Acute exacerbation of pancreatitis	2.566	0.955‒6.896	0.062			
Groove pancreatitis	2.050	0.271‒15.52	0.487			
Autoimmune pancreatitis	1.482	0.196‒11.23	0.703			
Parenchymal calcification	0.213	0.077‒0.586	0.003			
Pancreas atrophy	0.960	0.360‒2.561	0.935			
Pancreatic duct dilatation	1.602	0.456‒5.626	0.462			
Pancreatic duct stone	0.228	0.052‒1.005	0.051			
Pancreatic duct stricture	2.706	1.007‒7.271	0.048			
Irregular pancreatic duct	0.715	0.162‒3.150	0.658			
Pancreatic cyst	0.979	0.340‒2.818	0.969			
CBD stricture	2.112	0.767‒5.817	0.148			
Previous pancreatobiliary surgery	2.685	0.866‒8.326	0.087			
CA 19‒9 level (< 100 vs. ≥ 100 U/mL)	3.567	1.294‒9.831	0.014	3.528	1.282‒9.710	0.015

*HR* hazard ratio, *CI* confidence interval, *BMI* body mass index, *DM* diabetes mellitus.

### The incidence of pancreatic cancer according to risk factors

The incidence of pancreatic cancer per year was 0.49%, but the annual incidence of pancreatic cancer increased by using significant risk factors as 0.98% (age ≥ 60), 1.13% (no parenchymal calcification), 0.96% (pancreatic duct stricture), and 2.31% (CA 19‒9 ≥ 100 U/mL) (Table 5). When ≥ 60 years, no parenchymal calcification, and pancreatic duct stricture were combined, the annual incidence of pancreatic cancer increased up to 3.80%.

**Table 5:** Annual incidence of pancreatic cancer among patients with chronic pancreatitis according to risk factors.

No	Risk factors	N	Pancreatic cancer (n)	Overall incidence (%)	Annual incidence (%)
1	Age ≥ 60	292	12	4.11	0.98
2	No parenchymal calcification	215	10	4.65	1.13
3	Pancreatic duct stricture	152	7	4.61	0.96
4	CA 19‒9 ≥ 100 U/mL	54	5	9.26	2.31
1 + 2		103	7	6.80	1.74
2 + 3		69	6	8.70	2.23
1 + 3		56	5	8.93	2.35
1 + 2 + 3		31	4	12.90	3.80

## Discussion

CP is known as one of the major risk factors for pancreatic cancer. The SIRs of pancreatic cancer in patients with CP have been reported to be 7.6‒68.1^2–6^. The age- and sex-SIR in our study was 18.1 (95% CI 10.4‒29.5), which also demonstrated that CP has higher risk for pancreatic cancer.

There have been several studies on the incidence and relative risk of pancreatic cancer in patients with CP. The reported incidence of pancreatic cancer varied from study to study at 0.68‒2.94%^2,9,10,12,15^. There are many causes for this variation, and approaches to select patients with CP and the included CPs are the main cause. Studies using a large amount of data are thought to be more suitable for determining the incidence of pancreatic cancer in that these studies can analyze a large number of patients with CP. However, retrospective studies in which patients are selected by only the diagnostic ICD codes for CP are limited in accurately identifying CP patients^16^. Such studies can enroll patients with other pancreatic diseases, such as acute pancreatitis, pancreatic cancer, pancreas cystic neoplasm, and intraductal papillary mucinous neoplasm (IPMN), which may be frequently mistaken for CP. In addition, even for CP, it is sometimes not clear whether it is definite, probable, or borderline. Therefore, patients with various degrees of CP may be included in the studies. In this case, the incidence of pancreatic cancer can also be affected. In general, probable or borderline CP does not have clear diagnostic criteria, and the incidence of pancreatic cancer is not high in patients with probable or borderline CP compared to those with definite CP. Therefore, it is more desirable to select patients with definite CP and determine how many develop pancreatic cancer^7,11,12^. In our study, all imaging tests of the patients, as well as diagnostic codes, were reviewed to find definite CP. A few previous studies evaluated only patients who underwent surgery or endoscopic management for CP^3,9^. These patients had complicated CPs and were followed up well. However, they did not represent general CP, and surgery or endoscopic treatment can lead to a change in pancreas condition affecting the development of pancreatic cancer. In a multicenter retrospective study, patients who underwent surgery for the treatment of CP had significantly lower incidences of pancreatic cancer^5^. A recent published study in South Korea registered patients using only ICD-10 codes and reported a low pancreatic cancer incidence of 0.68%^15^. As observed in our study, a notable number of CP patients registered with only ICD code K86 might not have CP, and even if they had CP, the CP could be probable or borderline CP instead of definite CP. Thus, it was difficult to accurately identify the occurrence of pancreatic cancer in that study, and the incidence of pancreatic cancer would be lower than it really is.

Pancreatic cancer development time is another factor affecting the incidence of pancreatic cancer. Since both CP and pancreatic cancer can be found at the same time or pancreatic cancer can be mistaken for CP, newly developed pancreatic cancer in CP is usually defined as pancreatic cancer discovered 1‒2 years after the diagnosis of CP^17^. Previous studies had a different reference point of time; thus, interpreting these studies requires careful attention. In previous studies, the patient registration time was mainly at the time of outpatient visit, but in our study, patients were registered at the time when imaging studies confirmed pancreatic cancer-free CP. Therefore, it is considered rare for CP and pancreatic cancer to exist together or be mistaken for CP initially, and we set a 1-year washout period to reduce the confusion. In addition, our study showed that pancreatic cancer incidence remained relatively stable until 7 years. Pancreatic cancer and CP were diagnosed at the same time in 0.68% of CP patients, within a year in 0.41% of CP patients, at 1‒2 years in 0.69% of CP patients, at 2‒3 years in 0.71% of CP patients, at 3‒4 years in 0.70% of CP patients, at 4‒5 years in 0.30% of CP patients, at 5‒6 years in 0.78% of CP patients, and at 6‒7 years in 0.51% of CP patients. Since the incidence of pancreatic cancers remained steady over time and the incidence within 2 years was not significantly higher than that during other follow-up periods, very few or no cases of pancreatic cancer are considered to exist when CP was diagnosed. In a meta-analysis, the risk of pancreatic cancer was 6.09 after 1 year of diagnosis of CP, 16.16 after 2 years, 7.90 after 5 years, and 3.53 after 9 years, so the incidence of pancreatic cancer was high within approximately 5 years after diagnosis of CP^18^. Our study revealed that pancreatic cancer occurred within up to 6.5 years after diagnosis of CP. Based on the meta-analysis and our study, more active surveillance is needed up to 5‒6 years after diagnosis.

Although the incidence of pancreatic cancer is high in among patients with CP, surveillance of all patients with CP is not recommended because the incidence of pancreatic cancer itself is not high enough for surveillance. The 2020 International Consensus Guidelines also did not recommend screening all patients with CP for pancreatic cancer^14^. Therefore, it is recommended to select and monitor a subgroup of patients with CP with a higher risk of pancreatic cancer. International Consensus Guidelines recommend screening tests in patients with hereditary CP with *PRSS1* gene mutation. Unfortunately, the guidelines did not present other subgroups who should be monitored. Since the majority of CP cases are sporadic, not hereditary, it is necessary to identify which factors are associated with a high risk of pancreatic cancer in patients with sporadic CP and specify the subject for follow-up. The following risk factors for pancreatic cancer have been suggested: metabolic syndrome, *Helicobacter pylori* infection, obesity, alcohol use, and red meat consumption have a low risk (relative risk 1.1‒1.5); family history, long-term diabetes, and smoking have a moderate risk (relative risk 1.5‒2.0); and hereditary pancreatitis, germline mutation, and CP have a high risk (relative risk > 2.0)^14^. However, the risk in patients with CP is a lifetime risk and is not high on an annual basis. Therefore, it is necessary to determine subgroups with a high annual incidence of pancreatic cancer. Patients with newly developed diabetes are a high-risk group for pancreatic cancer, but similar to CP, screening for pancreatic cancer is not recommended for all new-onset diabetes patients. Therefore, it is necessary to know the subgroup that requires pancreatic cancer surveillance among patients with new-onset diabetes. A previous study identified groups for high-risk pancreatic cancer using the enrich new-onset diabetes for pancreatic cancer (ENDPAC) score model^19^. In the validation cohort including 1096 new-onset diabetes patients, 9 patients developed pancreatic cancers over 3 years (overall 0.82% and 0.27% per year), and 7 patients developed pancreatic cancers among the 197 patients with 3 or more ENPAC scores (overall 3.6% and 1.2% per year). Therefore, the study concluded that it is appropriate to monitor new-onset diabetes patients with ENPAC scores of 3 or higher. If this is applied to CP, selecting a subgroup with an annual incidence of pancreatic cancer of 1% or more would allow detect pancreatic cancer to be efficiently in patients with CP while reducing unnecessary tests and economic burdens. In our study, the incidence of pancreatic cancer was 0.49% per year in all CP patients; however, the annual incidences of pancreatic cancer in patients with significant risk factors, such as age greater than 60 years, no parenchymal calcification, pancreatic duct stricture, and CA 19‒9 levels greater than 100 U/mL increased the incidence of pancreatic cancer to 0.98%, 1.13%, 0.96%, and 2.31%, respectively, which is close to or greater than 1%. Even except for the CA 19‒9 level, if two or more factors were satisfied, the incidence of pancreatic cancer increased to 1.74‒2.35%, and if all three factors were satisfied, the incidence of pancreatic cancer was as high as 3.80%. Therefore, it is necessary to perform surveillance for CP subgroups that have each risk factor, and in particular, if two or more risk factors are satisfied, a more careful follow-up is needed.

There are some limitations in this study. First, the data were collected in a retrospective manner. Therefore, some data were missing or could not be analyzed. It is almost impossible to conduct a prospective study that requires a large number of patients with CP and long-term follow-up. Accordingly, all previous studies were performed in a retrospective manner. To reduce bias and ensure the quality of the data, we reviewed all patients' imaging studies. Second, we did not evaluate exocrine insufficiency due to a lack of available data on it, although it is included in the diagnostic criteria of definite CP. Additionally, the association between pancreatic cancer and lifestyle habits such as alcohol and tobacco use was not demonstrated due to the same reason. These should be supplemented by future research. Third, a few accompanied or confusing IPMN in the pancreas can be included in our study, since imaging studies cannot clearly distinguish between IPMN and CP. In some patients who underwent surgery, histologic findings revealed both IPMN and CP or IPMN instead of CP.

## Conclusion

Patients with CP had a high risk of developing pancreatic cancer. CP patients aged 60 years or older at CP diagnosis and with CA 19‒9 levels greater than 100 U/mL warrant careful surveillance for pancreatic cancer, and active surveillance is needed up to 5‒6 years after the diagnosis of CP.

## Methods

### Study population and data collection

Patients with ICD-10 codes for CP (K86.0, K86.1) who underwent abdominal CT or MRI between January 2010 and December 2021 in seven academic hospitals were selected using an electronic database: Clinical Data Warehouse in Catholic Medical Center, Seoul, South Korea. Data included basic demographic characteristics, follow-up times, CP or pancreatic cancer diagnosis time, CT or MRI findings, operation and histological findings, and laboratory results.

**Figure 4:**
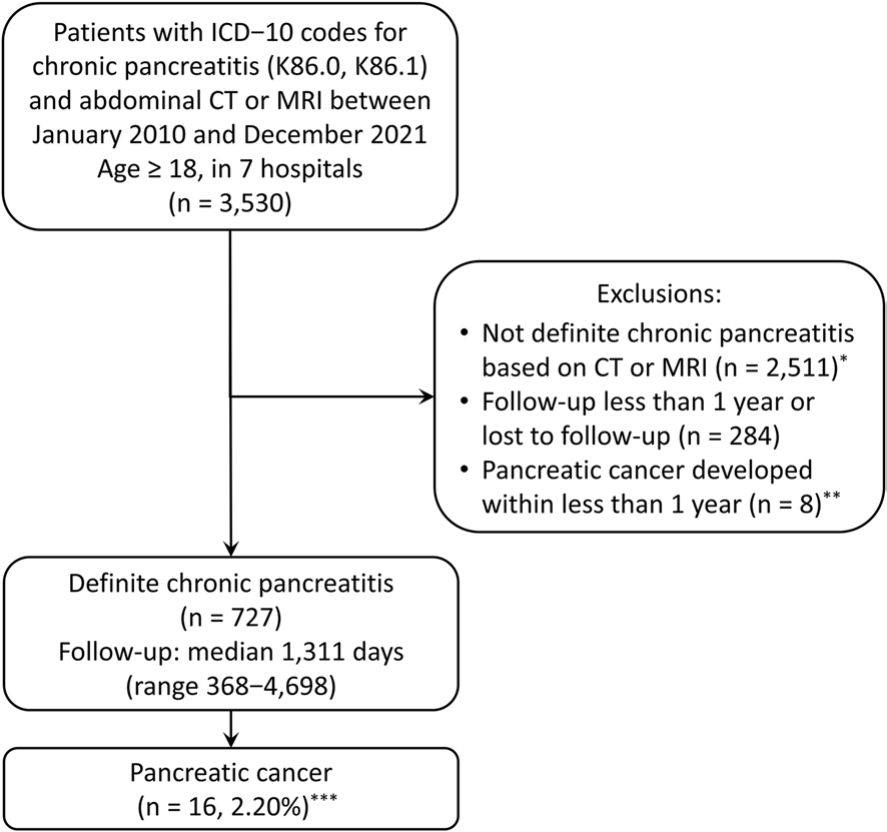
Study cohort. *Probable or borderline chronic pancreatitis, acute pancreatitis, pancreatic cancer, pancreatic neuroendocrine tumor, pancreas cystic neoplasm, pancreas solid pseudopapillary tumor, and ectopic pancreas, **Including five patients who had initial imaging findings of both pancreatic cancer and chronic pancreatitis, ***0.49% per year.

A total of 3530 patients older than 18 years were selected (Fig. 4). Patients who did not show definite CP by abdominal CT or MRI were excluded from the study (n = 2511), including those with probable or borderline CP, acute pancreatitis, pancreatic cancer, pancreatic neuroendocrine tumor, pancreas cystic neoplasm, pancreas solid pseudopapillary tumor, and ectopic pancreas. In addition, the patients who were not followed up for less than one year (n = 284) and those who developed pancreatic cancer within less than one year (n = 8) including five patients who presented pancreatic cancer and CP together initially were excluded. Finally, 727 patients with definite CP were analyzed.

### CP diagnostic criteria and definitions

The diagnosis of CP was based on the M-ANNHEIM diagnostic criteria, introduced in 2007^20^. In this criteria, definite CP consists of more than one of the following findings: pancreatic calcification, moderate or marked ductal lesions, marked and persistent exocrine insufficiency, and typical histology. Ductal changes were classified by the Cambridge classification. For the diagnosis of CP, CT or MRI findings and histology were reviewed, but pancreatic exocrine function was not referenced due to the lack of available data. Definite CPs were enrolled, and probable or borderline CPs were excluded from the study.

Age was determined at the time of the first imaging study with a diagnosis of CP. The diagnosis of pancreatic cancer was made when histologically confirmed. Newly developed pancreatic cancer was defined as a diagnosis of pancreatic cancer more than one year after being diagnosed with CP in an imaging study. The baseline serum CA 19‒9 levels were selected as the representative values. The start of the follow-up period was the time when the first diagnostic imaging test was performed, and the end of the follow-up period was the last contact with the patients or the time of pancreatic cancer diagnosis during follow-up.

### Ethics statement

Patient anonymity was carefully protected, and all study protocols were in complete compliance with the Declaration of Helsinki. This study obtained approval from the Institutional Review Board of Bucheon St. Mary's Hospital (No. HC21WIDI0125). Informed consent was not obtained due to the retrospective nature of the study. The documented informed consent was waived by decision of Institutional Review Board of Bucheon St. Mary's Hospital under the full understanding on the Clinical Data Warehouse search method of the present study.

### Statistical analysis

The results are reported using frequency (percent) or median (range or interquartile range) unless otherwise noted. Categorical and continuous data of clinical characteristics in the two groups were compared by Pearson’s chi-square test/Fisher’s exact test and Student’s t test/Mann-Whitney *U*-test. To calculate the standardized incidence ratio, the national cancer and population databases were utilized from the National Cancer Information Center and Korean Statistical Information Service. The cumulative incidence of pancreatic cancer was plotted using the Kaplan‒Meier method. Cox regression analyses were applied to determine risk factors related to the development of pancreatic cancer in patients with CP in univariate and multivariate analyses. The variables that were significant in the univariate analyses were used in the multivariate analysis. Considering the number of pancreatic cancer patients, multivariate analyses were repeatedly performed with two variables. The hazard ratios (HRs) with their 95% confidence intervals (CIs) were presented. A *p* value < 0.05 was considered to be significant. All statistical analyses were performed using SPSS (SPSS for Windows, version 20; Chicago, IL, USA).

## Data Availability

The datasets generated or analyzed during the current study are not publicly available due to privacy policies but are available from the corresponding author on reasonable request.
